# Commentary: Utility of EEG measures of brain function in patients with acute stroke

**DOI:** 10.3389/fnhum.2016.00621

**Published:** 2016-12-02

**Authors:** Brenton Hordacre, Nigel C. Rogasch, Mitchell R. Goldsworthy

**Affiliations:** ^1^School of Medicine, The Robinson Research Institute, The University of AdelaideAdelaide, SA, Australia; ^2^Department of Physiotherapy, Repatriation General Hospital, SA HealthDaw Park, SA, Australia; ^3^Brain and Mental Health Laboratory, School of Psychological Sciences and Monash Biomedical Imaging, Monash Institute of Cognitive and Clinical Neuroscience, Monash UniversityMelbourne, VIC, Australia; ^4^Discipline of Psychiatry, School of Medicine, The University of AdelaideAdelaide, SA, Australia

**Keywords:** electroencephalography, stroke, rehabilitation, impairment, transcranial alternating current stimulation, biofeedback

Several imaging and neurophysiological assessments are used to characterize both neural injury and neural function after stroke. These measures can inform clinical practice, map longitudinal changes, and guide therapeutic interventions. Electroencephalography (EEG) is one technique that measures neural function and can provide detailed assessment of spontaneous and task-related cortical oscillatory function. Neural oscillations reflect synchronized activity of large populations of cortical neurons which are fundamental for network communication and information processing. Although not currently a routine clinical assessment following stroke, EEG is a sensitive measure of cortical function and subsequent neural changes resulting from brain insults such as cerebral ischemia, and therefore has potential for wider clinical use.

Recently, Wu et al. (2016) investigated the capacity of EEG to capture behavioral impairment shortly following stroke. Resting state EEG recordings were performed in 25 patients between 3 and 12 days post-stroke in complex acute clinical settings. Using partial least squares (PLS) regression analysis, it was reported that delta power from a subset of electrodes predicted 72% of variance in acute stroke impairment measured with the National Institute of Health Stroke Scale, while beta power predicted 73% of variance (leave-one-out cross-validated). EEG coherence, a marker of functional connectivity, did not predict acute stroke impairment. Further, investigation of the PLS models revealed higher delta power in two regions, one overlying the ipsilesional sensorimotor cortex and one over the contralesional frontoparietal cortex, were associated with greater impairment. Similarly, reduced beta power in two regions, one overlying the ipsilesional primary motor cortex (M1) and the other over the contralesional parietal cortex, correlated with greater impairment.

Wu et al. (2016) reported several interesting findings which provide unique insight to acute stroke neurophysiology. Here, we highlight those key findings and discuss their significance in advancing the field. First, the finding that abnormalities in spectral power, but not coherence, were related to acute post-stroke impairment is an interesting observation in light of recent findings in chronic stroke. Using a similar PLS approach, EEG recorded in chronic stroke survivors identified that beta frequency coherence between M1 and ipsilesional motor networks was a strong predictor of motor impairment and recovery of function (Wu et al., 2015). Why similar relationships were not observed in the acute post-stroke period is not clear. It may be that acute neural damage following an ischemic lesion causes rapid changes in synchronization of the local neural network, affecting functional output. As the motor network reorganizes across the sub-acute post-stroke period, different neural populations may be recruited in order to restore function, meaning the strength and flexibility of motor network connectivity would become an important marker of function and capacity for further recovery (Park et al., 2011). Nevertheless, these results suggest power is an early marker of stroke impairment, while the importance of connectivity may increase during sub-acute or chronic post-stroke periods.

An important application of EEG is the ability to investigate neural oscillations in specific frequency bands. Insight into the functional significance of different frequency oscillations may provide an additional source of neurophysiological information. For example, previous stroke studies suggest delta oscillations originate from the region of the obstructed cerebral artery, reflecting reduced regional cerebral blood flow (Finnigan et al., 2006; Finnigan and van Putten, 2013). Furthermore, restoration of cerebral blood flow following administration of a tissue plasminogen activator was associated with normalization of delta power within minutes (Finnigan et al., 2006). Wu et al. (2016) report a positive correlation between ipsilesional delta power and infarct volume which appears to support previous findings indicating a relationship between delta power and cerebral blood flow following ischemic stroke. However, the relationship between beta power and impairment may reflect the importance of beta oscillations to motor function. Beta oscillations are associated with motor preparation and output and have been recorded over motor network regions during movement (Wheaton et al., 2005). Furthermore, beta coherence in sensorimotor regions recorded at rest was found to be a strong predictor of motor learning (Wu et al., 2014). However, the relationships between abnormalities in beta power and impairment following stroke are unclear. It may be that reduced beta power and impairment are both driven by neuronal loss following ischemic stroke. Investigating causal relationships between power and impairment represents an important progression in determining the clinical utility of EEG and may direct further interventional studies to facilitate greater functional recovery following stroke. Transcranial alternating current stimulation (tACS) has been shown to entrain neural oscillations in a frequency specific manner (Zaehle et al., 2010), resulting in subsequent behavioral change (Pollok et al., 2015). Similarly, biofeedback allows participants to control frequency specific neural rhythms and has been used to modulate spectral power (Mulholland, 1995). Following on from the findings of Wu et al. (2016), an important progression would be to employ these neuromodulatory techniques to determine if changing EEG power has an effect on motor recovery following stroke. Such studies may advance acute stroke care by informing novel interventional approaches capable of improving post-stroke brain function and reducing impairment. While speculative and requiring further investigation, if abnormalities in beta power are related to post-stroke impairment, beta frequency tACS or biofeedback could be used as interventional techniques to normalize beta power recorded over the ipsilesional M1 and contralesional parietal cortex. Such interventions may assist restitution of motor function and represent an important progression in stroke recovery.

Future studies investigating causal relationships between EEG measures and impairment should also consider approaches to improve the spatial specificity of scalp EEG. Volume conduction is a significant limitation for interpreting the anatomical location of neural oscillatory activity. Several analytical techniques can increase the spatial specificity of oscillatory power and connectivity analyses, such as using spatial filtering methods (e.g., Laplacian re-referencing or source localization; Nunez et al., 1997; Schoffelen and Gross, 2009) or measures of connectivity which are less sensitive to volume conduction than coherence (e.g., imaginary coherence or weighted phase lag index; Nolte et al., 2004; Vinck et al., 2011). However, the field is still advancing these methods, and thus, results need to be treated carefully.

In summary, Wu et al. (2016) report several interesting neurophysiological observations following acute stroke. To further advance this field of research, future studies should investigate causal relationships between neural function and impairment using techniques such as tACS and biofeedback. These approaches may help decipher the clinical utility of EEG and inform future interventional studies which may lead to improved clinical outcomes.

## Author contributions

BH, NR, and MG all made substantial intellectual contributions to the manuscript and approved the final version for publication.

## Funding

NR is supported by a research fellowship from the National Health and Medical Research Council of Australia (1072057). MG is supported by an NHMRC-ARC Dementia Research Development Fellowship (1102272).

### Conflict of interest statement

The authors declare that the research was conducted in the absence of any commercial or financial relationships that could be construed as a potential conflict of interest.
